# Training Needs and Quality of Public Health Inspectors in Greece during the COVID-19 pandemic: Demetris Lamnisos

**DOI:** 10.1093/eurpub/ckac131.373

**Published:** 2022-10-25

**Authors:** I Adamopoulos, D Lamnisos, N Syrou, G Boustras

**Affiliations:** 1 Department of Health Sciences, European University Cyprus, Nicosia, Cyprus; 2 School of Sciences, European University Cyprus, Nicosia, Cyprus; 3 Department of Physical Education and Sport, University of Thesally, Thesally, Greece; 4 CERIDES, European University Cyprus, Nicosia, Cyprus

## Abstract

**Background:**

Public Health Inspectors (PHI) task is to promote health, respond to emerging threats in Public Health and prevent and control of the communicable and environmental diseases. During the COVID-19 pandemic, PHI and other Healthcare Workers faced greater biological risks of infections and therefore personal protection was of great importance for their health and safety. Staff education and training is an important factor of disaster management capability and may help prevent and control pandemics. This study took place during the COVID-19 pandemic and aimed to investigate the training needs and training quality of Greek Public Health Inspectors.

**Methods:**

A nationwide cross-sectional study was conducted between March and June 2021 in Greece. An online survey was distributed to respondents by email through the National Public Health Inspectorate Administration. The survey included seven Likert scale items for assessing the training needs (health and safety of work, stress management, health services administration, crisis management in the health sector, natural disasters, personal protective equipment and protection against biological agents and protection from chemical agents) and two Likert scale items for assessing the training quality.

**Results:**

The response rate was 30.5 % (185/606 PHI). The 62.2% were females with a mean age of 49 years old (sd = 8.2) and a mean work experience of 15.9 years (sd = 8.5). Most participants (64.51%) reported high training needs on health and safety at work, stress management and personal protective equipment. Most participants (43.78%) also reported low training quality. Training needs were higher for women and in urban and semi-urban workplace environments.

**Conclusions:**

High training needs and low rate in training quality are important issues for PHI in all workplace environments. PHI need training in health and safety at work, stress management and personal protective equipment.

**Key messages:**

• High training needs and low rate in training quality are important issues for Public Health Inspectors in all workplace environments in Greece.

• Future training for Greek Public Health Inspectors should focus on health and safety at work, stress management and personal protective equipment.

